# Assessing SSDoH in Alzheimer's Research: Core Measures and Those in Sexual and Gender Minority Populations

**DOI:** 10.1093/geroni/igab046.304

**Published:** 2021-12-17

**Authors:** Shana Stites, Sharnita Midgett

**Affiliations:** 1 University of Pennsylvania, Philadelphia, Pennsylvania, United States; 2 University Pennsylvania, Philadelphia, Pennsylvania, United States

## Abstract

Social and structural determinants of health (SSDoH) are conditions that can impact on Alzheimer’s disease and Alzheimer’s disease related dementias (AD/ADRD) outcomes. We will describe theoretical underpinnings of core SSDoH constructs and their measure, empirical evidence for their importance, and their potential for elucidating disease and prevention mechanisms. We focus on a core set of SSDoH measures that are important across a broad range of socially and culturally heterogeneous populations. We outline a rationale for universal implementation of a set of SSDoH measures and juxtapose the approach with alternatives, such as investigator-initiated grants, aimed at collecting SSDoH data. We also speak very briefly about the evidence supporting assessing SSDoH with respect to sex, gender, and sexual orientation and considerations in doing this.

